# Coagulation phenotypes in sepsis and effects of recombinant human thrombomodulin: an analysis of three multicentre observational studies

**DOI:** 10.1186/s13054-021-03541-5

**Published:** 2021-03-19

**Authors:** Daisuke Kudo, Tadahiro Goto, Ryo Uchimido, Mineji Hayakawa, Kazuma Yamakawa, Toshikazu Abe, Atsushi Shiraishi, Shigeki Kushimoto

**Affiliations:** 1 Division of Emergency and Critical Care Medicine, Tohoku University Graduate School of Medicine, 1-1 Seiryo-machi, Aoba-ku, Sendai, 980-8574 Japan; 2 Department of Clinical Epidemiology and Health Economics, School of Public Health, The University of Tokyo, 7-3-1 Hongo, Bunkyo-ku, Tokyo, 113-0033 Japan; 3 Intensive Care Unit, Tokyo Medical and Dental University Medical Hospital, 1-5-45 Yushima, Bunkyo-ku, Tokyo, 113-8519 Japan; 4 Department of Emergency Medicine, Hokkaido University Hospital, Kita 14 Nishi-5, Kita-ku, Sapporo, 060-8648 Japan; 5 Division of Emergency Medicine, Osaka Medical College, 2-7 Daigakumachi, Takatsuki, 569-8686 Japan; 6 Department of Emergency and Critical Care Medicine, Tsukuba Memorial Hospital, 1187-299 Kaname, Tsukuba, 300-2622 Japan; 7 Health Services Research and Development Center, University of Tsukuba, 1-1-1 Tennodai, Tsukuba, 305-8577 Japan; 8 Emergency and Trauma Center, Kameda Medical Center, 929 Higashimachi, Kamogawa, 296-8602 Japan

**Keywords:** Anticoagulants, Disseminated intravascular coagulation, Machine learning, Phenotype, Precision medicine, Thrombomodulin

## Abstract

**Background:**

A recent randomised trial showed that recombinant thrombomodulin did not benefit patients who had sepsis with coagulopathy and organ dysfunction. Several recent studies suggested presence of clinical phenotypes in patients with sepsis and heterogenous treatment effects across different sepsis phenotypes. We examined the latent phenotypes of sepsis with coagulopathy and the associations between thrombomodulin treatment and the 28-day and in-hospital mortality for each phenotype.

**Methods:**

This was a secondary analysis of multicentre registries containing data on patients (aged ≥ 16 years) who were admitted to intensive care units for severe sepsis or septic shock in Japan. Three multicentre registries were divided into derivation (two registries) and validation (one registry) cohorts. Phenotypes were derived using *k*-means with coagulation markers, platelet counts, prothrombin time/international normalised ratios, fibrinogen, fibrinogen/fibrin-degradation-products (FDP), D-dimer, and antithrombin activities. Associations between thrombomodulin treatment and survival outcomes (28-day and in-hospital mortality) were assessed in the derived clusters using a generalised estimating equation.

**Results:**

Four sepsis phenotypes were derived from 3694 patients in the derivation cohort. Cluster dA (*n* = 323) had severe coagulopathy with high FDP and D-dimer levels, severe organ dysfunction, and high mortality. Cluster dB had severe disease with moderate coagulopathy. Clusters dC and dD had moderate and mild disease with and without coagulopathy, respectively. Thrombomodulin was associated with a lower 28-day (adjusted risk difference [RD]: − 17.8% [95% CI − 28.7 to − 6.9%]) and in-hospital (adjusted RD: − 17.7% [95% CI − 27.6 to − 7.8%]) mortality only in cluster dA. Sepsis phenotypes were similar in the validation cohort, and thrombomodulin treatment was also associated with lower 28-day (RD: − 24.9% [95% CI − 49.1 to − 0.7%]) and in-hospital mortality (RD: − 30.9% [95% CI − 55.3 to − 6.6%]).

**Conclusions:**

We identified four coagulation marker-based sepsis phenotypes. The treatment effects of thrombomodulin varied across sepsis phenotypes. This finding will facilitate future trials of thrombomodulin, in which a sepsis phenotype with high FDP and D-dimer can be targeted.

**Supplementary Information:**

The online version contains supplementary material available at 10.1186/s13054-021-03541-5.

## Background

The global rate of sepsis-related mortality remains high and the annual age-standardised mortality owing to sepsis is 148.1 deaths per 100,000 of the global population [1]. Recombinant human thrombomodulin (rhTM) has anti-inflammatory and anticoagulation activities [2], and it has been suggested as an adjunct therapy for patients with sepsis, particularly those with sepsis-induced coagulopathy [3]. Nevertheless, a recent phase III randomised controlled trial revealed no beneficial effect of rhTM in patients with sepsis-induced coagulopathy [4]. This result can be explained by the heterogeneity of patients with sepsis and inappropriate criteria of coagulopathy [5] using the prothrombin time/international normalised ratio (PT-INR) and a platelet count based on subgroup analysis of an international phase II trial of rhTM [6].

Sepsis is a highly heterogeneous syndrome with variable aetiology and pathophysiology [7]. Thus, a specific therapy may benefit some, but not all, patients with sepsis. Several recent studies have classified sepsis into several phenotypes with distinct characteristics using cluster analysis [8–10], an unsupervised machine learning method that can identify relatively homogeneous groups in a heterogeneous population [11]. Furthermore, these studies indicated that specific therapies conferred benefits only in patients with specific phenotypes of sepsis [8–10].

Identifying the target patients receptive to rhTM treatment through grouping based on biomarker cut-offs is challenging. To address this issue, we examined latent sepsis phenotypes in terms of coagulopathy and identified which phenotypes would benefit from rhTM using machine learning approaches.

## Methods

### Study design and settings

Details of the methods and analytical processes in the present study are provided in the Supplemental Digital Content. This was a secondary analysis of the following multicentre registries: the Japan Septic Disseminated Intravascular Coagulation (JSEPTIC-DIC) study (UMIN-CTR ID: UNIN000012543) [12], Tohoku Sepsis Registry (UMIN000010297) [13], and Focused Outcomes Research in Emergency Care for Acute Respiratory Distress Syndrome, Sepsis, and Trauma (FORECAST) sepsis study (UMIN000019742) [14]. All three registries include information on consecutive patients admitted to ICUs for severe sepsis or septic shock [15, 16]. Briefly, the JSEPTIC-DIC study retrospectively reviewed data derived from 3195 consecutive patients with severe sepsis or septic shock, aged ≥ 16 years, admitted to 42 ICUs at 40 institutions in Japan, between January 2011 and December 2013[12]. The Tohoku Sepsis Registry prospectively registered 616 consecutive patients who were admitted to ICUs with severe sepsis, or those who developed severe sepsis after admission to the ICUs or general wards at 10 institutions (three university hospitals and seven community hospitals) in the Tohoku District, Northern Japan, between January 2015 and December 2015 [13]. The multicentre prospective FORECAST sepsis study included 1184 consecutive patients aged ≥ 16 years, who were admitted to 59 ICUs in Japan with severe sepsis according to the sepsis-2 criteria [15], between January 2016 and March 2017 [14]. These studies were approved and the need for informed consent was waived by the institutional review boards at the participating hospitals.

### Study population

We included all patients (aged ≥ 16 years), who were admitted to the ICUs with severe sepsis or septic shock as defined in the three registries, according to the International Sepsis Definitions Conference criteria [15, 16].

### Phenotyping variables

We measured the following coagulation markers upon admission to the ICU for phenotyping: platelet counts, PT-INR, fibrinogen, fibrinogen/fibrin degradation products (FDP), D-dimer, and antithrombin activities.

### Exposure

Patients were exposed to rhTM.

### Outcomes

The main outcomes were 28-day and in-hospital mortality in the validation cohort. The secondary outcomes were ICU-free days, ventilator-free days, and the type of discharge in the validation cohort.

### Definitions

Ventilator-free days were defined as the number of days on which a patient did not require mechanical ventilation during the initial 28 days following enrolment. The number of ventilator-free days of patients, who died within day 28, was assigned as 0. ICU-free days were calculated similarly.

### Statistical analysis

#### Analytical cohorts

We derived sepsis phenotypes from the JSEPTIC-DIC study (*n* = 3195) and Tohoku Sepsis Registry (*n* = 499) and validated the phenotypes using the FORECAST sepsis study (*n* = 1184).

#### Cluster derivation

We initially assessed the distribution and missingness in phenotyping variables. Non-normal data were log-transformed and scaled. Patients without 28-day mortality information were excluded. Missing data were imputed using the random forest method for each study cohort with the *missForest* package [17]. Random forest imputation is a nonparametric algorithm that accommodates nonlinearities and interactions and does not require the specification of a specific parametric model [18]. This approach generated single-point estimates by random draws from independent normal distributions centred on conditional means predicted by random forest. Random forest applies bootstrap aggregation of multiple regression trees to reduce the risk of overfitting and combines estimates from many trees [17]. Missingness was imputed using patient characteristics, laboratory data, outcomes, and other covariates, including in-hospital management (Additional file 1: Supplemental documents).

We applied *k*-means with Euclid distance, which is a basic and widely used machine learning-based clustering approach, to derive sepsis phenotypes [9, 11]. We then determined the optimal number of clusters using a consensus clustering approach that assessed both quantitatively and visually to estimate the number of unsupervised classes in a dataset by inducing sampling variability with sub-sampling [19]. In consensus clustering, we evaluated the separation of consensus matrix heatmaps using the elbow method, cumulative distribution function, and cluster-consensus plots. We visually evaluated the clustering using t-Distributed Stochastic Neighbour Embedding (t-SNE) for reducing dimensionality and visualising high-dimensional datasets [20]. We also derived phenotypes using a divisive hierarchical clustering approach as an alternative to *k*-means, for confirming the cluster consistency. The number of clusters was determined using the dendrogram and the elbow and gap statistic methods [21].

#### Evaluation of rhTM effects in derived phenotypes

We examined the associations between rhTM and the clinical outcomes in each derived cluster, using a generalised estimating equation to adjust for hospital-level variance. We analysed the associations after adjusting for the potential confounders of age, sex, comorbidities, and sequential organ failure assessment (SOFA) scores. In the derivation cohort, we did not adjust for the management before and after admission to the hospitals because the information on when each management was initiated was not available. We examined the cluster-level effects of rhTM by including the interaction term, rhTM use –x– cluster, in the model to examine different effects of rhTM across clusters. In addition, to confirm the robustness of the association of interest, we applied a Bayesian regression model to assess the associations between rhTM and the clinical outcomes for each derived cluster, based on *k*-means in the derivation cohort [21, 22]. Bayesian regression was performed using a Markov chain Monte Carlo procedure with four chains of 2000 iterations per chain. The results are shown as beta coefficients with 95% credible intervals and displayed as odds ratios with 95% credible intervals for simplicity.

#### Cluster validation and evaluation of rhTM effects

We predicted patient phenotypes in the FORECAST sepsis study as external data based on coagulation markers of clusters in the derivation cohort (derived from JSEPTIC-DIC and Tohoku Sepsis Registry). Predictions arose from the Euclidean distance from each patient to the centroid of each FORECAST phenotype. In each predicted cluster in the FORECAST sepsis study, we first described the frequency and clinical characteristics of the clusters. Thereafter, we used a generalised estimating equation to account for patient clustering within hospitals to assess associations between rhTM and clinical outcomes in each predicted cluster in the external data. The adjusted variables were age, sex, comorbidities, SOFA scores, and in-hospital management, including renal replacement therapy, and treatment with steroids, intravenous immunoglobulin, antithrombin, and vasopressors. As the FORECAST sepsis data included information on the time of management, we included management before or after admission as a covariate to estimate the effects of rhTM on clinical outcomes. For sensitivity analyses, we used a generalised estimating equation, applying the acute physiology and chronic health evaluation (APACHE II) score and source of infection as adjusted variables, instead of the SOFA score. The source of infection was categorised into respiratory, abdominal, skin and soft tissue, urinary tract, and others. As in the derivation cohort, we analysed the association by Bayesian regression with a Markov chain Monte Carlo procedure with four chains.

Values with *p* < 0.05 were considered statistically significant. All data were analysed using Stata version 14.1 (StataCorp, College Station, TX, USA) and R version 3.4.1 package for t-SNE (https://cran.r-project.org/web/packages/tsne/tsne.pdf) (R Foundation, Vienna, Austria).

## Results

### Patients in the derivation cohort

We excluded 117 patients without 28-day mortality information in the derivation cohort from the two multicentre registries, leaving 3694 patients who were eligible for analysis (3195 from JSEPTIC-DIC and 499 from the Tohoku Sepsis Registry). Table 1 summarises the patients’ characteristics. The median age was 72 years, and 40% of patients were female. Overall, rhTM was administered to 26.2% of patients (the distribution of the proportion of patients in the institutes is shown in Additional file 3: Figure S1). The in-hospital mortality and 28-day mortality rates were 32.1% and 20.4%, respectively.

**Table 1:** Characteristics of patients in the derivation cohort according to clusters

	Overall	Cluster dA	Cluster dB	Cluster dC	Cluster dD	P*
Variables	*n* = 3694	*n* = 323	*n* = 629	*n* = 1147	*n* = 1595	
Age*, median (IQR)	72.0 (62.0, 81.0)	72.0 (58.0, 80.0)	72.0 (63.0, 81.0)	73.0 (63.0, 81.0)	72.0 (62.0, 80.0)	0.25
Sex*, female	1468 (39.7%)	164 (50.8%)	268 (42.6%)	483 (42.1%)	553 (34.7%)	< 0.001
Body weight kg, median (IQR)	54.7 (46.6, 64.2)	55.0 (47.5, 64.0)	52.0 (45.0, 61.1)	55.0 (46.8, 64.3)	55.0 (47.0, 65.0)	< 0.001
Comorbidity*						
Liver	149 (4.0%)	28 (8.7%)	73 (11.6%)	30 (2.6%)	18 (1.1%)	< 0.001
Respiratory	141 (3.8%)	8 (2.5%)	23 (3.7%)	43 (3.7%)	67 (4.2%)	0.52
Cardiovascular	316 (8.6%)	20 (6.2%)	49 (7.8%)	97 (8.5%)	150 (9.4%)	0.23
Renal	306 (8.3%)	24 (7.4%)	50 (7.9%)	111 (9.7%)	121 (7.6%)	0.23
Immunodeficiency	709 (19.2%)	62 (19.2%)	119 (18.9%)	233 (20.3%)	295 (18.5%)	0.69
Infection site						< 0.001
Unknown	218 (6.8%)	32 (11.0%)	49 (8.4%)	64 (6.4%)	73 (5.5%)	
Catheter-related	44 (1.4%)	1 (0.3%)	6 (1.0%)	19 (1.9%)	18 (1.4%)	
Bone/soft tissue	374 (11.7%)	20 (6.9%)	60 (10.3%)	108 (10.9%)	186 (14.0%)	
Cardiovascular	68 (2.1%)	13 (4.5%)	4 (0.7%)	26 (2.6%)	25 (1.9%)	
Central nervous system	63 (2.0%)	14 (4.8%)	1 (0.2%)	26 (2.6%)	22 (1.7%)	
Urinary tract	509 (15.9%)	71 (24.5%)	40 (6.8%)	210 (21.1%)	188 (14.2%)	
Lung/thoracic	827 (25.9%)	38 (13.1%)	117 (20.0%)	243 (24.4%)	429 (32.3%)	
Abdomen	1032 (32.3%)	94 (32.4%)	294 (50.3%)	279 (28.1%)	365 (27.5%)	
Other	60 (1.9%)	7 (2.4%)	13 (2.2%)	19 (1.9%)	21 (1.6%)	
APACHE II, median (IQR)	22.0 (17.0, 28.0)	26.0 (20.0, 33.0)	26.0 (19.0, 32.0)	23.0 (17.0, 29.0)	20.0 (15.0, 26.0)	< 0.001
SIRS score, median (IQR)	3.0 (2.0, 4.0)	3.0 (3.0, 4.0)	3.0 (2.0, 4.0)	3.0 (2.0, 4.0)	3.0 (2.0, 4.0)	< 0.001
SOFA scores*	9.0 (6.0, 12.0)	13.0 (10.0, 16.0)	11.0 (9.0, 14.0)	10.0 (7.0, 12.0)	7.0 (5.0, 10.0)	< 0.001
Lab data						
White blood cell (10^3^/μL), median (IQR)	11.3 (4.8, 17.8)	12.2 (4.6, 19.7)	7.8 (2.2, 15.5)	11.7 (6.0, 18.6)	11.6 (6.0, 17.5)	< 0.001
**Platelet (**10^3^/μL), median (IQR)	122.0 (65.0, 194.0)	59.5 (32.0, 92.0)	78.0 (46.5, 128.0)	103.0 (54.0, 162.0)	178.0 (121.0, 252.0)	< 0.001
**PT-INR**, median (IQR)	1.3 (1.2, 1.6)	1.6 (1.4, 2.1)	1.7 (1.5, 2.2)	1.3 (1.2, 1.5)	1.2 (1.1, 1.4)	< 0.001
**Fibrinogen (**mg/mL), median (IQR)	421.0 (296.0, 528.9)	231.0 (151.0, 311.0)	245.3 (157.0, 350.0)	452.0 (367.0, 563.0)	476.9 (395.3, 576.0)	< 0.001
**FDP (**μg/mL), median (IQR)	17.6 (10.1, 36.2)	120.2 (79.2, 266.0)	16.0 (10.4, 24.0)	34.3 (22.8, 55.1)	10.0 (7.6, 13.8)	< 0.001
**D-dimer (**μg/mL), median (IQR)	7.8 (3.9, 17.2)	51.9 (35.2, 113.0)	7.7 (4.8, 11.7)	15.4 (10.5, 25.0)	3.8 (2.7, 5.6)	< 0.001
**Antithrombin (**%), median (IQR)	60.0 (50.8, 69.0)	52.0 (42.4, 60.5)	42.6 (33.0, 50.4)	60.1 (54.0, 68.0)	66.0 (59.0, 73.7)	< 0.001
Lactate (mmol/L), median (IQR)	2.9 (1.7, 5.7)	5.3 (2.9, 10.1)	4.3 (2.3, 8.0)	2.7 (1.5, 5.4)	2.3 (1.4, 4.1)	< 0.001
ISTH DIC score						< 0.001
0	685 (18.7%)	0 (0.0%)	15 (2.4%)	25 (2.2%)	645 (41.1%)	
1	239 (6.5%)	2 (0.6%)	17 (2.7%)	15 (1.3%)	205 (13.0%)	
2	701 (19.2%)	3 (0.9%)	104 (16.6%)	116 (10.2%)	478 (30.4%)	
3	592 (16.2%)	25 (7.8%)	99 (15.8%)	327 (28.8%)	141 (9.0%)	
4	530 (14.5%)	42 (13.0%)	143 (22.8%)	261 (23.0%)	84 (5.3%)	
5	441 (12.1%)	79 (24.5%)	105 (16.7%)	240 (21.1%)	17 (1.1%)	
6	250 (6.8%)	78 (24.2%)	83 (13.2%)	88 (7.7%)	1 (0.1%)	
7	169 (4.6%)	72 (22.4%)	38 (6.1%)	59 (5.2%)	0 (0.0%)	
8	40 (1.1%)	20 (6.2%)	15 (2.4%)	5 (0.4%)	0 (0.0%)	
ISTH DIC score ≥ 5	1430 (39.2%)	291 (90.7%)	384 (62.0%)	653 (57.5%)	102 (6.5%)	
Managements						
rhTM	969 (29.3%)	128 (44.1%)	184 (31.5%)	334 (33.6%)	210 (15.8%)	< 0.001
Vasopressor use	2789 (75.5%)	289 (89.5%)	558 (88.7%)	882 (76.9%)	1060 (66.5%)	< 0.001
Renal replacement therapy	971 (26.3%)	135 (41.8%)	220 (35.0%)	339 (29.6%)	277 (17.4%)	< 0.001
Steroids	894 (24.2%)	112 (34.7%)	214 (34.1%)	285 (24.8%)	283 (17.7%)	< 0.001
Intravenous immunoglobulin	1088 (29.5%)	116 (35.9%)	239 (38.0%)	362 (31.6%)	371 (23.3%)	< 0.001
Antithrombin	1092 (29.6%)	161 (49.8%)	296 (47.1%)	367 (32.0%)	268 (16.8%)	< 0.001
Outcomes						
28-day death	753 (20.4%)	117 (36.2%)	198 (31.5%)	200 (17.4%)	238 (14.9%)	< 0.001
In-hospital death	1186 (32.1%)	151 (46.8%)	301 (47.9%)	358 (31.0%)	376 (23.6%)	< 0.001

Six coagulation markers (bold font) were used for clustering

*APACHE* acute physiology and chronic health evaluation, *DIC* disseminated intravascular coagulation, *FDP* fibrinogen/fibrin degradation product, *IQR* interquartile range, *PT-INR* prothrombin time-international normalised ratio, *SIRS* systemic inflammatory response syndrome, *SOFA* sequential organ failure assessment, *WBC* white blood cells

Variables with asterisk (*) were potential confounders that were adjusted in a generalised estimating equation. *P between clusters

### Derivation of clinical sepsis phenotypes

**Fig. 1:**
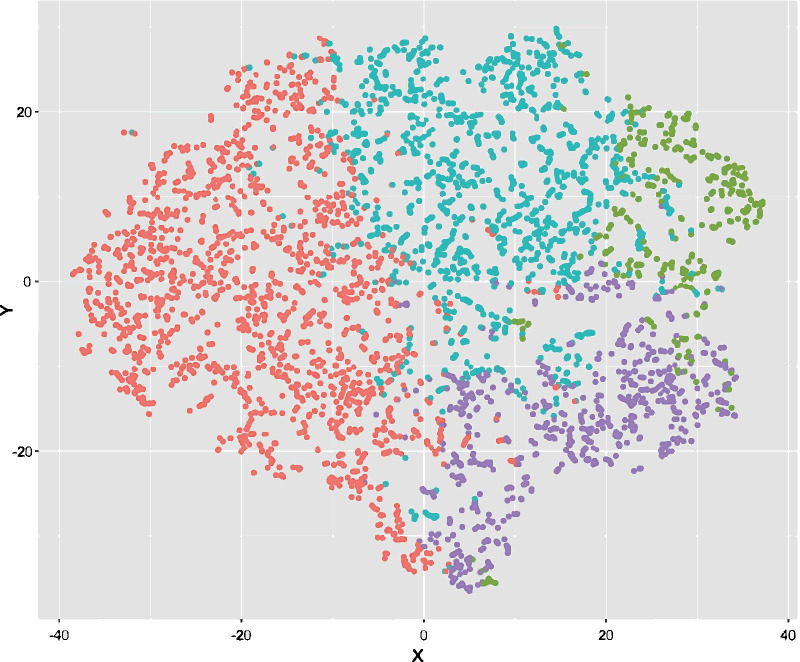
t-SNE plot. This t-Distributed Stochastic Neighbour Embedding (t-SNE) plot is a dimensionality reduction technique for graphically simplifying extensive datasets. Four clusters are plotted, and some patients are on the borderlines between clusters. Circles represent individual patients (green, cluster dA; purple, cluster dB; blue, cluster dC; red, cluster dD)

We assessed the distributions and missingness among the phenotyping variables. Antithrombin activity was the most lacking variable, 51.3% and 45.8% in derivation and validation datasets, respectively (Table 2). According to clustering using *k*-means, a four-class model including the phenotype clusters derivation dA, dB, dC, and dD (“d” represents “derivation”) may be an optimal fit. The heatmap matrix (Additional file 3: Figure S2), elbow method (Additional file 3: Figure S3), and cumulative distribution function curve (Additional file 3: Figure S4), indicated that the four-class model was optimal, whereas the cluster-consensus plot suggested that two, three, or four clusters were optimal (Additional file 3: Figure S5). The four-class model was supported by the t-SNE plot with clear separation (Fig. 1). Additional file 3: Figure S6 shows a cluster dendrogram obtained using a divisive hierarchical clustering approach. The elbow method showed that a two- or four-cluster model is optimal (Additional file 3: Figure S7), whereas the gap statistic method [21] showed that the four-cluster model was optimal (Additional file 3: Figure S8).

**Table 2:** Missingness in phenotyping variables based on study cohorts

Variables	JSEPTIC-DIC (*n* = 3195)	Tohoku sepsis registry (*n* = 499)	FORECAST (*n* = 1184)
	*n* (%)	*n* (%)	*n* (%)
Platelets	10 (0.3%)	74 (14.8%)	6 (0.5%)
PT-INR	187 (5.9%)	155 (31.1%)	38 (3.2%)
Fibrinogen	753 (23.6%)	269 (53.9%)	226 (19.1%)
FDP	1101 (34.5%)	243 (48.7%)	376 (31.8%)
D-dimer	839 (26.3%)	220 (44.1%)	301 (25.4%)
Antithrombin activity	1550 (48.5%)	346 (69.3%)	542 (45.8%)

*FDP* fibrinogen/fibrin degradation product, *PT-INR* prothrombin time-international normalised ratio

Patients in cluster dA were likely to have a severe physiological status and organ dysfunction (high APACHE II and SOFA scores), coagulopathy (low platelet counts, prolonged PT-INR, low fibrinogen, and extremely high FDP and D-dimer levels), high lactate levels, and high mortality (Table 1). Approximately 90% of patients in this cluster required vasopressors. The characteristics of patients in cluster dB were similar to that in cluster dA in terms of severity, but likely to have abdominal infection with normal white blood cell counts, moderate coagulopathy with moderate FDP and D-dimer levels, and low antithrombin activity. Patients in clusters dC and dD had moderate and mild disease, respectively. Although patients in cluster dC had coagulopathy with high FDP and D-dimer levels, those in cluster dD were likely to have respiratory infection without coagulopathy. The phenotypes were similar according to the four-cluster hierarchical clustering (Additional file 2: Table S1).

### Evaluation of rhTM effects in the derivation cohort

Recombinant human thrombomodulin was administered to 128 (44.1%), 184 (31.5%), 334 (33.6%), and 210 (15.5%) patients in clusters dA, dB, dC, and dD, respectively. Clinical outcomes in cluster dA were better with than in those without rhTM (adjusted risk difference [RD], − 17.8% [95% CI, − 28.7 to − 6.9%] for 28-day mortality; RD, − 17.7% [95% CI − 27.6 to − 7.8%] for in-hospital mortality (Table 3). Analysis of the rhTM effect modification across clusters using cluster dA as the reference showed that the effects of rhTM differed across clusters (all, *p* < 0.05), except for in-hospital mortality in cluster dB (*p* = 0.31). The associations were similar according to the four-cluster hierarchical clustering (Additional file 2: Table S2). Furthermore, rhTM treatment was associated with better clinical outcomes in cluster dA according to Bayesian regression (Additional file 2: Table S3).

**Table 3:** Unadjusted and adjusted risk difference between recombinant thrombomodulin use and outcomes

Outcomes	Cluster dA	*p* value	Cluster dB	*p* value	Cluster dC	*p* value	Cluster dD	*p* value
Associations in the derivation cohorts, risk difference, % (95%CI)								
Unadjusted association (vs. non rhTM use)								
28-Day death	− 10.8 (− 21.5 to − 0.1)	0.047	3.5 (− 4.6 to 11.5)	0.40	− 1.9 (− 6.9 to 3.2)	0.47	2.2 (− 2.8 to 7.1)	0.39
In-hospital death	− 10.9 (− 20.8 to − 1.1)	0.03	1.6 (− 7.2 to 10.3)	0.73	− 8.0 (− 13.8 to − 2.3)	0.01	0.8 (− 5.2 to 6.8)	0.78
Adjusted association (vs. non rhTM use)								
28-Day death	− 17.8 (− 28.7 to − 6.9)	0.001	0.7 (− 7.1 to 8.6)	0.85	− 3.1 (− 8.3 to 2.1)	0.24	− 0.7 (− 4.5 to 6.0)	0.79
In-hospital death	− 17.7 (− 27.6 to − 7.8)	< 0.001	0.2 (− 7.9 to 8.3)	0.97	− 10.2 (− 15.9 to − 4.6)	< 0.001	− 1.3 (− 7.6 to 4.9)	0.67

	Cluster vA	*p* value	Cluster vB	*p* value	Cluster vC	*p* value	Cluster vD	*p* value
Associations in the validation cohorts, risk difference, % (95%CI)								
Unadjusted association (vs. non-rhTM use)								
28-Day death	− 15.0 (− 32.2 to 2.2)	0.09	3.2 (− 12.5 to 18.9)	0.69	4.4 (− 5 to 13.8)	0.36	7.1 (− 2.4 to 16.6)	0.14
In-hospital death	− 22.2 (− 39.6 to − 4.9)	0.01	8.8 (− 7.3 to 24.8)	0.29	5.7 (− 4.9 to 16.2)	0.3	14.2 (3.8 to 24.7)	0.008
Adjusted association (vs. non rhTM use)								
28-Day death	− 24.9 (− 49.1 to − 0.7)	0.04	− 5.7 (− 29.9 to 18.5)	0.64	1.4 (− 12.8 to 15.7)	0.84	− 6.7 (− 19.4 to 6.0)	0.3
In-hospital death	− 30.9 (− 55.3 to − 6.6)	0.01	− 3.7(− 27.9 to 20.5)	0.77	− 0.5 (− 16.1 to 15.1)	0.95	0.7 (− 13.0 to 14.5)	0.92

In the derivation cohort, the adjusted variables were age, sex, comorbidities, and sequential organ failure assessment (SOFA) scores

In the validation cohort, the adjusted variables were age, sex, comorbidities, SOFA scores, and in-hospital management, including renal replacement therapy, and treatment with steroids, intravenous immunoglobulin, antithrombin, and vasopressors

*rhTM* recombinant human thrombomodulin

### Characteristics of phenotypes in the validation cohort

Additional file 2: Table S4 shows the patients’ characteristics in each cluster in the validation cohort. The median age was 73 years, 40% of the patients were women, and rhTM was administered to 21.2% of patients. In-hospital and 28-day mortality rates were 23.4% and 19.0%, respectively. These characteristics were similar to those in the derivation cohort but the rate of rhTM treatment and mortality were relatively lower.

We used only coagulation markers to predict clusters in the validation cohort, and the characteristics were similar to those in the derivation cohort (“v” represents “validation”). Similar to the patients in cluster dA, those in cluster vA were likely to have a severe physiological status and organ dysfunction (high APACHE II and SOFA scores), coagulopathy (low platelet counts, prolonged PT-INR, low fibrinogen, and extremely high FDP and D-dimer levels), high lactate levels, and moderately high mortality. Patients in cluster vB had a high mortality rate with moderate coagulopathy and moderate FDP and D-dimer levels. Patients in clusters vC and vD had moderate and mild disease, respectively. Patients in cluster vC had coagulopathy with high FDP and D-dimer levels, whereas those in cluster vD did not have coagulopathy.

### Evaluation of the effects of rhTM in the validation cohort

All 1184 patients in the FORECAST sepsis study dataset were analysed for validation. Recombinant human thrombomodulin was administered to 44 (44.4%), 54 (31.2%), 98 (26.3%), and 46 (9.3%) patients in clusters vA, vB, vC, and vD, respectively. Clinical outcomes in cluster vA were better than in those without rhTM (adjusted RD, − 24.9% [95% CI − 49.1 to − 0.7%] for 28-day mortality; RD − 30.9% [95% CI − 55.3 to − 6.6%] for in-hospital mortality; Table 3). In contrast, rhTM was not associated with better outcomes in the other clusters. As secondary outcomes, rhTM was associated with increased number of ventilator-free days (6.7 days [95% CI 0.8–12.7 days]) in Cluster vA, but not with the number of ICU-free days or discharge location in any of the clusters (Additional file 2: Table S5). In the sensitivity analyses, rhTM was associated with better outcomes, when APACHE II score and source of infection were applied as adjusted variables, instead of the SOFA score (adjusted RD, − 0.24% [95% CI − 0.45 to − 0.02%] for 28-day mortality; RD − 0.26% [95% CI − 0.47 to − 0.04%] for in-hospital mortality; Additional file 2: Table S6). The associations between rhTM, and 28-day and in-hospital mortalities were consistent with the findings of the Bayesian regression analysis (Additional file 2: Table S3 and Additional file 3: Figure S9).

## Discussion

This secondary analysis of the sepsis registries identified four phenotypes with various coagulation features among patients with severe sepsis. Treatment with rhTM was associated with lower in-hospital mortality rates only in the phenotype with severe coagulopathy, characterised by low platelet counts, extremely high levels of FDP and D-dimer (phenotype clusters dA and vA), and severe organ dysfunction. These results were not identified in the other phenotypes.

The severity of coagulopathy is defined by the DIC scoring systems, such as the International Society on Thrombosis and Haemostasis (ISTH) scoring system for diagnosing overt DIC [23] and Japanese Association for Acute Medicine DIC scoring system [24], both of which have been applied in many studies. The difference between these systems and machine learning-based clustering is the use of a trivial cut-off. Table 1, Additional file 2: Tables S1, and S4 show that each phenotype cluster included patients with various ISTH DIC scores without a clear cut-off that overlapped with the other clusters. This suggests that clustering based on machine learning can detect novel phenotypes that cannot be identified using the conventional scoring systems.

Recombinant human thrombomodulin has anticoagulation effects and was shown to be beneficial for patients with sepsis and coagulopathy in observational studies and in a subgroup analysis of a phase II trial [3, 6]. The latest phase III trial focused on patients with sepsis with cardiovascular or respiratory dysfunction as well as coagulopathy according to subgroup analysis of the phase II trial [4]. However, the phase III trial did not identify a positive effect of rhTM on survival, suggesting that differentiating a subgroup that may benefit from rhTM is difficult using conventional methods with clear cut-offs. In our study, despite overlapping characteristics, various DIC scores, and differences in severity among clusters, cluster vA (dA) was the only phenotype in which rhTM was associated with better survival outcomes. This suggests that machine learning clustering can identify optimal clinical phenotypes for rhTM treatment. Additionally, the machine learning clustering described herein used only six variables, all of which are general markers that can be measured in most hospitals. Additionally, the results can be available soon after admission before deciding to administer rhTM in an emergency room or ICU.

Other studies using machine learning-based clustering for patients with sepsis also suggest that several specific therapies have beneficial effects only in patients with specific phenotypes. A Toll-like receptor 4 antagonist, protocol-based resuscitation, activated protein C, and fluid input affected each phenotype differently [9, 10]. The effectiveness of rhTM also varied across phenotypes in our study. Therefore, selecting an optimal clinical phenotype may be key to the success of a specific therapy for patients with sepsis. Including entire populations with sepsis may explain why previous randomised trials found no beneficial effects of adjunctive therapies [25–28]. The goal of precision/tailored medicine is to select the optimal therapy for patients, for which machine learning-based clustering can be effective. Although our study does not fully address the definite endotypes of coagulation in sepsis biologically or pathophysiologically, our findings improve the understanding of the true endotypes of sepsis with coagulation.

### Limitations

This study had several limitations. We used three registries that included different variables. Therefore, unmeasured confounders, and a lack of information such as the timing of rhTM administration may have biased our findings. Nevertheless, the data included detailed clinical information that is generally used for adjustment, and the results in the validation cohort accounted for management before or after admission. The number of missing variables for clustering was not small; therefore, missingness may have limited our findings. However, missing data imputation using the random forest approach is considered valid and valid imputation reduces bias, even when the proportion of missingness is high [29]. Our data did not include information on the long-term outcomes (i.e. 6-/12-months mortality) and SOFA score at several weeks after admission, although long-term outcomes are also important. Our data did not include the duration of rhTM administration, which was presumably 6 days, according to the generally prescribed dose and duration in Japan. We could not evaluate whether the phenotypes and efficacy of rhTM are applicable for patients with sepsis defined by the Sepsis-3 criteria [30], as three observational studies enrolled patients with sepsis using the Sepsis-2 definition [16], and the datasets did not include SOFA scores before admission. Heparin is commonly used for anticoagulant therapy, worldwide. However, we could not include heparin treatment for sepsis-induced coagulopathy in the model, because two of three registries did not collect the data. Only 5% (167/3195) of the patients were treated with heparin for coagulopathy with sepsis (excluding use for venous thromboembolism) in JSEPTIC-DIC study [12]. We need to develop a model to determine the phenotypes of individual patients to be able to perform a clinical trial in the future. Finally, our data were derived from Japanese patients; therefore, the generalisability of the results may be limited.

## Conclusions

The findings derived using machine learning clustering indicated that rhTM can benefit only patients with a severe coagulopathy phenotype. Identifying patients for whom a therapy will have a beneficial effect can lead to precision/tailored medicine in critical care. To achieve this goal, the accuracy of phenotyping should be increased by analysing more patients, and through further validation. A randomised trial focusing on suitable phenotypes determined by effective phenotyping is warranted.

### Supplementary Information

**Additional file 1.** Supplemental documents.

**Additional file 2.** Supplemental Tables.

**Additional file 3.** Supplemental Figures.

## Data Availability

The datasets generated and/or analysed during the original studies are available in the Scientific data, https://www.nature.com/articles/sdata2018243 (J-SEPTIC DIC study), and Mendeley Data, https://data.mendeley.com/datasets/vvv89kw3k5/1 (Tohoku Sepsis Registry). The dataset of the FORECAST sepsis study is not publicly available, based on the decision by the Japanese Association for Acute Medicine.

## Appendix: The core investigators FORECAST Study

Satoshi Gando 1, 2), Yasuhiro Otomo 3), Shigeki Kushimoto 4), Hiroshi Ogura 5), Seitaro Fujishima 6), Atsushi Shiraishi 7), Daizoh Saitoh 8), Toshihiko Mayumi 9), Kiyotsugu Takuma 10), Taka-aki Nakada 11), Yasukazu Shiino 12), Takehiko Tarui 13), Toru Hifumi 14), Kohji Okamoto 15), Yutaka Umemura 16), Joji Kotani 17), Yuichiro Sakamoto 18), Junichi Sasaki 19), Shin-ichiro Shiraishi 20), Ryosuke Tsuruta 21), Akiyoshi Hagiwara 22), Toshikazu Abe 23, 24, 25), Kazuma Yamakaw 26), Tomohiko Masuno 27), Naoshi Takeyama 28), Norio Yamashita 29), Hiroto Ikeda 30), Masashi Ueyama 31)

1) Division of Acute and Critical Care Medicine, Hokkaido University, Sapporo, Japan

2) Critical Care Medicine, Sapporo Higashi Tokushukai Hospital, Sapporo, Japan

3) Trauma and Acute Critical Care Center, Medical Hospital, Tokyo Medical and Dental University, Tokyo, Japan

4) Division of Emergency and Critical Care Medicine, Tohoku University Graduate School of Medicine, Sendai, Japan

5) Department of Traumatology and Acute Critical Medicine, Osaka University Graduate School of Medicine, Osaka, Japan

6) Center for General Medicine Education, Keio University School of Medicine, Tokyo, Japan

7) Emergency and Trauma Center, Kameda Medical Center, Kamogawa, Japan

8) Division of Traumatology, Research Institute, National Defense Medical College, Tokorozawa, Japan

9) Department of Emergency Medicine, School of Medicine, University of Occupational and Environmental Health, Kitakyushu, Japan

10) Emergency & Critical Care Center, Kawasaki Municipal Kawasaki Hospital, Kawasaki, Japan

11) Department of Emergency and Critical Care Medicine, Chiba University Graduate School of Medicine, Chiba, Japan

12) Department of Acute Medicine, Kawasaki Medical School, Kurashiki, Japan

13) Department of Trauma and Critical Care Medicine, Kyorin University School of Medicine, Tokyo, Japan

14) Department of Emergency and Critical Care Medicine, St. Luke’s International Hospital, Tokyo, Japan

15) Department of Surgery, Center for Gastroenterology and Liver Disease, Kitakyushu City Yahata Hospital, Kitakyushu, Japan

16) Department of Disaster and Emergency Medicine, Kobe University Graduate School of Medicine, Kobe, Japan

17) Department of Traumatology and Acute Critical Medicine, Osaka University Graduate School of Medicine, Osaka, Japan

18) Emergency and Critical Care Medicine, Saga, University Hospital, Saga, Japan

19) Department of Emergency and Critical Care Medicine, Keio University School of Medicine, Tokyo, Japan

20) Department of Emergency and Critical Care Medicine, Aizu Chuo Hospital, Aizuwakamatsu, Japan

21) Advanced Medical Emergency & Critical Care Center, Yamaguchi, Ube, Japan

22) Department of Emergency Medicine, Niizashiki Chuo General Hospital, Niiza, japan

23) Department of General Medicine, Juntendo University, Tokyo, Japan

24) Health Services Research and Development Center, University of Tsukuba, Tsukuba, Japan

25) Department of Health Services Research, Faculty of Medicine, University of Tsukuba, Tsukuba, Japan

26) Division of Trauma and Surgical Critical Care, Osaka, General Medical Center, Osaka, Japan

27) Department of Emergency and Critical Care Medicine, Nippon Medical School, Tokyo, Japan

28) Advanced Critical Care Center, Aichi Medical University Hospital, Nagakute, Japan

29) Advanced Emergency medical Service Center, Kurume University Hospital, Kurume, Japan

30) Department of Emergency Medicine, Teikyo University School of Medicine, Tokyo, Japan

31) Department of Trauma, Critical Care Medicine, and Burn Center, Japan Community Healthcare Organization, Chukyo Hospital

## Abbreviations

APACHE: Acute physiology and chronic health evaluation
FDP: Fibrinogen/fibrin degradation product
FORECAST: Focused Outcomes Research in Emergency Care for Acute Respiratory Distress Syndrome, Sepsis, and Trauma
ICU: Intensive care unit
ISTH: International Society on Thrombosis and Haemostasis
JSEPTIC-DIC: Japan Septic Disseminated Intravascular Coagulation
PT-INR: Prothrombin time/international normalised ratio
RD: Risk difference
rhTM: Recombinant human thrombomodulin
SOFA: Sequential organ failure assessment
