# Supplementary material for: Coagulation phenotypes in sepsis and effects of recombinant human thrombomodulin: an analysis of three multicentre observational studies
Source: Crit Care. 2021 Mar 19;25:114. doi: 10.1186/s13054-021-03541-5 (PMC7978458; doi:10.1186/s13054-021-03541-5)
Supplement: Supplementary file 1 — Additional file 1. Supplemental documents. [file 13054_2021_3541_MOESM1_ESM.docx]

**Supplemental documents**

**Coagulation Phenotypes in Sepsis and Effects of Recombinant Human Thrombomodulin: An Analysis of Three Multicentre Observational Studies**

Authors: Daisuke Kudo, Tadahiro Goto, Ryo Uchimido, Mineji Hayakawa, Kazuma Yamakawa, Toshikazu Abe, Atsushi Shiraishi, Shigeki Kushimoto

**Details on the methods**

**Study design and settings**

This secondary analysis used the Tohoku Sepsis Registry (UMIN-CTR ID: UMIN000010297), the sepsis registries in the Japan Septic Disseminated Intravascular Coagulation (JSEPTIC-DIC; UNIN000012543), and the Focused Outcomes Research in Emergency Care for Acute Respiratory Distress Syndrome, Sepsis, and Trauma FORECAST sepsis (UMIN000019742) studies. Briefly, the Tohoku Sepsis Registry prospectively registered 616 consecutive patients who were admitted to ICU with severe sepsis, or who developed severe sepsis after admission to an ICU or general wards at 10 institutions (three university hospitals and seven community hospitals) in the Tohoku District, Northern Japan between January 2015 and December 2015 (1). The JSEPTIC-DIC study retrospectively reviewed data derived from 3,195 consecutive patients aged ≥16 years at 40 institutions admitted to 42 ICU between with severe sepsis or septic shock January 2011 and December 2013 (2). The multicentre prospective FORECAST sepsis study involved 1,184 consecutive patients aged ≥16 years who were admitted to 59 ICU in Japan with severe sepsis according to the sepsis-2 criteria (3) between January 2016 and March 2017 (4). These three studies were registered ion the University Hospital Medical Information Network Clinical Trials Registry and approved by the institutional review boards at each hospital. The boards waived the requirement for informed consent. According to Japanese law, no ethical approval was required for innominate available data.

**Study population**

We included all patients (aged ≥16 years) who were admitted to ICU for severe sepsis or septic shock defined in the three registries according to the International Sepsis Definitions Conference criteria (5).

**Exposure**

Exposure comprised the administration of rhTM regardless of timing and duration. We administered 380 U/kg/d rhTM to patients with disseminate intravascular coagulation (DIC) caused by any disease but without severe renal dysfunction, 130 U/kg/d to patients with severe renal dysfunction according to the generally prescribed dose and duration in Japan.

**Outcomes**

The main outcomes were 28-day and in-hospital mortality in the validation cohort. The secondary outcomes were ICU-free days, ventilator-free days, and the type of discharge in the validation cohort.

**Definitions**

Ventilator-free days were defined as the number of days on which a patient did not require mechanical ventilation during the initial 28 days following enrolment. The number of ventilator free days of patients, who died within day 28, was assigned as 0. ICU-free days were calculated similarly.

**Statistical analysis**

*Analytical cohorts*

We derived sepsis phenotypes from the JSEPTIC-DIC study (n = 3,195) and the Tohoku Sepsis Registry (n = 499) and validated them using FORECAST sepsis study (n = 1,184). Phenotyping using machine learning requires a large sample size; therefore, we derived the phenotypes from the JSEPTIC-DIC and Tohoku datasets.

*Phenotyping variables*

The following coagulation markers were measured upon admission to the ICU for phenotyping; platelet counts, prothrombin time/international normalized ratio (PT-INR), fibrinogen, fibrinogen/fibrin-degradation-products (FDP), D-dimer, and antithrombin activity levels. These markers were selected based on rhTM anticoagulation activities (6, 7), and on observational studies that found an association between rhTM and better outcomes among patients who had sepsis with coagulopathy (8, 9).

*Cluster derivation*

We initially assessed the distribution and missingness of phenotyping variables (**Table 2**). Non-normal data were log transformed and scaled. Patients without 28-day mortality data were excluded. Missing data were imputed by the random forest method for each study cohort using the *missForest* package (10). Random forest imputation is a nonparametric algorithm that accommodates nonlinearities and interactions and does not require a specific parametric model (11). Using this approach, single-point estimates were generated by random draws from independent normal distributions centred on conditional means predicted by random forest, which uses the bootstrap aggregation of multiple regression trees to reduce risk of overfitting and combines estimates from many trees (10). Missingness was imputed using patient characteristics (age, sex, weight), severity of the sepsis during admission (acute physiology and chronic health evaluation [APACHE] II score, each component of sequential organ failure assessment [SOFA] score, systemic inflammatory response syndrome [SIRS] score), comorbidities (cardiac, pulmonary, liver, renal, immunologic), laboratory test results on admission (white blood cells, haemoglobin, PT-INR, FDP, fibrinogen, d-dimer, antithrombin, lactate), treatments (rhTM, antithrombin, protease inhibitors, steroids, immunoglobulins, continuous renal replacement therapy, polymyxin B-immobilized fibre column hemoperfusion therapy) and outcomes (28-day mortality, ICU mortality, and length of hospital stay).

We derived sepsis phenotypes by applying k-means with Euclidean distance, which is a basic, widely used machine learning-based clustering approach (12, 12). We then determined the optimal number of clusters by consensus clustering, which provides quantitative and visual stability evidence with which to estimate the number of unsupervised classes in a dataset by inducing sampling variability with sub-sampling (14). We evaluated data generated in separation of consensus matrix heatmaps, the elbow method, cumulative distribution function, and cluster-consensus plots. We also visually evaluated clustering using t-Distributed Stochastic Neighbor Embedding (t-SNE), which reduces dimensionality and is particularly suited for visualizing high-dimensional datasets (15). We derived phenotypes using a divisive hierarchical clustering approach as an alternative to k-means. In this analysis, to confirm cluster consistency, the number of clusters was decided using dendrogram, elbow, and gap statistics (16).

*Evaluation of rhTM effects on the derived phenotypes*

We used a generalized estimating equation to adjust for hospital-level variance and assess associations between rhTM administration and clinical outcomes in the derived clusters. Although patients within a derived cluster presumably had similar characteristics in terms of rhTM medication, we analysed these associations after adjusting for the potential confounders of age, sex, comorbidities, and SOFA scores. We did not adjust for management before and after admission to the hospitals in the derivation cohort because no information was available about the time management was initiated. We also included the interaction term, rhTM use –x– cluster, in the model to determine the effect modification of rhTM at the cluster level. A significant interaction term indicated different effects of rhTM across clusters. To confirm the association of interest robustness, we applied a Bayesian regression model to assess associations between rhTM and clinical outcomes for each derived cluster based on k-means in the derivation cohort (17). Bayesian regression was achieved using a Markov Chain Monte Carlo procedure with four chains, and 2,000 iterations per chain. Results are shown as beta coefficients with 95% credible intervals. For simplicity, we displayed odds ratios (OR) with 95% credible intervals.

*Cluster validation and evaluation of rhTM effects*

In the FORECAST sepsis study, as external data,

We predicted patient phenotypes in the FORECAST sepsis study as external data based on the coagulation markers of clusters in the derivation cohort (that is, the JSEPTIC-DIC and Tohoku Sepsis Registry). Predictions arose from the Euclidean distance from each patient to the centroid of each FORECAST phenotype. In each predicted cluster in the FORECAST sepsis study, we first described the frequency and clinical characteristics of the clusters. We then applied a generalized estimating equation to account for patient clustering within hospitals to assess associations between rhTM and clinical outcomes in each predicted cluster in the external data. The adjusted variables were age, sex, comorbidities, SOFA scores, and in-hospital management, including renal replacement therapy, and treatment with steroid, intravenous immunoglobulin, antithrombin, and vasopressor. Because the FORECAST sepsis data included information about the time of management, we included the management before and after admission to the hospitals as a covariate to estimate the effect of rhTM on clinical outcomes. For sensitivity analyses, we used a generalized estimating equation, applying the acute physiology and chronic health evaluation (APACHE II) score and source of infection as adjusted variables, instead of the SOFA score. The source of infection was categorized into respiratory, abdominal, skin and soft tissue, urinary tract, and others. We analysed the associations using Bayesian regression with a Markov chain Monte Carlo procedure with four chains, similar to that used for the derivation cohort.

Values with *p* < 0.05 were considered statistically significant. All data were statistically analysed using Stata version 14.1 (StataCorp, College Station, TX, USA) and R version 3.4.1 package for t-SNE (tsne) (<https://cran.r-project.org/web/packages/tsne/tsne.pdf>) (R Foundation, Vienna, Austria).

**Availability of data and material**

J-SEPTIC DIC study: <https://www.nature.com/articles/sdata2018243>

Tohoku Sepsis Registry: <https://data.mendeley.com/datasets/vvv89kw3k5/1>

FORECAST sepsis study: not applicable

**Code availability**

R version 3.4.1 package for t-SNE (‘tsne’) (<https://cran.r-project.org/web/packages/tsne/tsne.pdf>) (R Foundation, Vienna, Austria).

**References**

1. Kudo D, Kushimoto S, Miyagawa N, Sato T, Hasegawa M, Ito F, et al. The impact of organ dysfunctions on mortality in patients with severe sepsis: A multicentre prospective observational study. J Crit Care 2018; 45: 178-83.

2. Hayakawa M, Yamakawa K, Saito S, Uchino S, Kudo D, Iizuka Y, et al. Nationwide registry of sepsis patients in Japan focused on disseminated intravascular coagulation 2011-2013. Sci Data 2018; 5: 180243.

3. Levy MM, Fink MP, Marshall JC, Abraham E, Angus D, Cook D, et al. 2001 SCCM/ESICM/ACCP/ATS/SIS International Sepsis Definitions Conference. Intensive Care Med 2003; 29: 530-8.

4. Abe T, Ogura H, Shiraishi A, Kushimoto S, Saitoh D, Fujishima S, et al. Characteristics, management, and in-hospital mortality among patients with severe sepsis in intensive care units in Japan: the FORECAST study. Crit Care 2018; 22: 322.

5. Dellinger RP, Levy MM, Rhodes A, Annane D, Gerlach H, Opal SM, et al. Surviving sepsis campaign: international guidelines for management of severe sepsis and septic shock: 2012. Crit Care Med 2013; 41: 580-637.

6. Abeyama K, Stern DM, Ito Y, Kawahara K, Yoshimoto Y, Tanaka M, et al. The N-terminal domain of thrombomodulin sequesters high-mobility group-B1 protein, a novel antiinflammatory mechanism. Journal Clin Invest 2005; 115: 1267-74.

7. Gomi K, Zushi M, Honda G, Kawahara S, Matsuzaki O, Kanabayashi T, et al. Antithrombotic effect of recombinant human thrombomodulin on thrombin-induced thromboembolism in mice. Blood 1990; 75: 1396-9.

8. Hayakawa M, Yamakawa K, Saito S, Uchino S, Kudo D, Iizuka Y, et al. Recombinant human soluble thrombomodulin and mortality in sepsis-induced disseminated intravascular coagulation. A multicentre retrospective study. Thromb Haemost 2016; 115: 1157-66.

9. Yamakawa K, Ogura H, Fujimi S, Morikawa M, Ogawa Y, Mohri T, et al. Recombinant human soluble thrombomodulin in sepsis-induced disseminated intravascular coagulation: a multicentre propensity score analysis. Intensive Care Med 2013; 39: 644-52.

10. Stekhoven DJ, Bühlmann P. MissForest--non-parametric missing value imputation for mixed-type data. Bioinformatics 2012; 28: 112-8.

11. Shah AD, Bartlett JW, Carpenter J, Nicholas O, Hemingway H. Comparison of random forest and parametric imputation models for imputing missing data using MICE: a CALIBER study. Am J Epidemiol 2014; 179: 764-74.

12. Seymour CW, Kennedy JN, Wang S, Chang CH, Elliott CF, Xu Z, et al. Derivation, validation, and potential treatment implications of novel clinical phenotypes for sepsis. JAMA 2019; 321: 2003-17.

13. Steinley D. K-means clustering: a half-century synthesis. Br J Math Stat Psychol 2006; 59: 1-34.

14. Wilkerson MD, Hayes DN. ConsensusClusterPlus: a class discovery tool with confidence assessments and item tracking. Bioinformatics 2010; 26: 1572-3.

15. Hinton G. Visualizing Data using t-SNE. J Mach Learn Res 2008; 9: 2579-605.

16. Tibshirani R, Walther G, Hastie T. Estimating the number of clusters in a data set via the gap statistic. J R Stat Soc Series B Stat Methodol 2001; 63: 411-23.

17. Zampieri FG, Costa EL, Iwashyna TJ, Carvalho CRR, Damiani LP, Taniguchi LU, et al. Heterogeneous effects of alveolar recruitment in acute respiratory distress syndrome: a machine learning reanalysis of the Alveolar Recruitment for Acute Respiratory Distress Syndrome Trial. Br J Anaesth 2019; 123: 88-95.
