# Supplementary material for: Coagulation phenotypes in sepsis and effects of recombinant human thrombomodulin: an analysis of three multicentre observational studies
Source: Crit Care. 2021 Mar 19;25:114. doi: 10.1186/s13054-021-03541-5 (PMC7978458; doi:10.1186/s13054-021-03541-5)
Supplement: Supplementary file 2 — Additional file 2. Supplemental Tables. [file 13054_2021_3541_MOESM2_ESM.docx]

**Supplemental tables**

**Table S1. Characteristics of patients in validation cohort clustered using divisive hierarchical clustering.**

|  | Overall | Cluster dA-hc | Cluster dB-hc | Cluster dC-hc | Cluster dD-hc | *P** |
| --- | --- | --- | --- | --- | --- | --- |
| Variables | n = 3694 | n = 609 | n = 602 | n = 1094 | n = 1389 |  |
| Age, median (IQR) | 72.0 (62.0, 81.0) | 72.0 (61.0, 80.0) | 71.0 (61.0, 80.0) | 72.0 (63.0, 80.0) | 73.0 (63.0, 81.0) | 0.053 |
| Sex, female | 1468 (39.7%) | 277 (45.5%) | 252 (41.9%) | 389 (35.6%) | 550 (39.6%) | <0.001 |
| Body weight kg, median (IQR) | 54.7 (46.6, 64.2) | 55.0 (48.0, 65.0) | 54.0 (46.0, 63.0) | 55.0 (47.0, 65.0) | 54.5 (45.9, 64.0) | 0.092 |
| Comorbidities |  |  |  |  |  |  |
| Liver | 149 (4.0%) | 30 (4.9%) | 62 (10.3%) | 37 (3.4%) | 20 (1.4%) | <0.001 |
| Respiratory | 141 (3.8%) | 21 (3.4%) | 18 (3.0%) | 46 (4.2%) | 56 (4.0%) | 0.58 |
| Cardiovascular | 316 (8.6%) | 43 (7.1%) | 43 (7.1%) | 111 (10.1%) | 119 (8.6%) | 0.078 |
| Renal | 306 (8.3%) | 52 (8.5%) | 49 (8.1%) | 80 (7.3%) | 125 (9.0%) | 0.50 |
| Immunodeficiency | 709 (19.2%) | 105 (17.2%) | 142 (23.6%) | 210 (19.2%) | 252 (18.1%) | 0.019 |
| Infection site |  |  |  |  |  | <0.001 |
| Unknown | 218 (6.8%) | 45 (8.3%) | 61 (11.1%) | 55 (5.8%) | 57 (5.0%) |  |
| Catheter-related | 44 (1.4%) | 7 (1.3%) | 11 (2.0%) | 11 (1.2%) | 15 (1.3%) |  |
| Bone/soft tissue | 374 (11.7%) | 60 (11.0%) | 57 (10.4%) | 131 (13.7%) | 126 (11.0%) |  |
| Cardiovascular | 68 (2.1%) | 18 (3.3%) | 15 (2.7%) | 13 (1.4%) | 22 (1.9%) |  |
| Central nervous system | 63 (2.0%) | 23 (4.2%) | 10 (1.8%) | 9 (0.9%) | 21 (1.8%) |  |
| Urinary tract | 509 (15.9%) | 142 (26.1%) | 85 (15.5%) | 122 (12.8%) | 160 (13.9%) |  |
| Lung/thoracic | 827 (25.9%) | 81 (14.9%) | 114 (20.8%) | 290 (30.4%) | 342 (29.8%) |  |
| Abdomen | 1032 (32.3%) | 158 (29.0%) | 183 (33.3%) | 318 (33.3%) | 373 (32.5%) |  |
| Other | 60 (1.9%) | 11 (2.0%) | 13 (2.4%) | 5 (0.5%) | 31 (2.7%) |  |
| APACHE2, median (IQR) | 22.0 (17.0, 28.0) | 23.0 (18.0, 30.0) | 26.0 (19.0, 33.0) | 20.0 (15.0, 26.0) | 22.0 (16.0, 27.0) | <0.001 |
| SIRS scores, median (IQR) | 3.0 (2.0, 4.0) | 3.0 (3.0, 4.0) | 3.0 (2.0, 4.0) | 3.0 (2.0, 4.0) | 3.0 (2.0, 4.0) | 0.003 |
| SOFA scores | 9.0 (6.0, 12.0) | 1.0 (0.0, 3.0) | 1.0 (1.0, 3.0) | 1.0 (0.0, 2.0) | 1.0 (0.0, 3.0) | <0.001 |
| Laboratory data |  |  |  |  |  |  |
| WBC (10^3^/μL), median (IQR) | 11.3 (4.8, 17.8) | 12.8 (6.3, 19.8) | 9.1 (2.9, 16.1) | 9.8 (3.6, 15.3) | 12.6 (7.2, 19.1) | <0.001 |
| **Platelets (**10^3^/μL), median (IQR) | 122.0 (65.0, 194.0) | 78.0 (42.0, 137) | 50.0 (31.0, 76.0) | 123 (79.0, 191) | 180 (134.0, 252) | <0.001 |
| **PT-INR**, median (IQR) | 1.3 (1.2, 1.6) | 1.4 (1.2, 1.7) | 1.5 (1.3, 1.9) | 1.3 (1.1, 1.6) | 1.3 (1.1, 1.5) | <0.001 |
| **Fibrinogen (**mg/mL), median (IQR) | 421 (296, 529) | 373 (254, 502) | 318(198, 457) | 428 (308, 520) | 468 (375, 567) | <0.001 |
| **FDP (**μg/mL), median (IQR) | 17.6 (10.1, 36.2) | 85.4 (62.4, 138.2) | 26.7 (18.7, 38.3) | 8.3 (6.6, 11.0) | 17.8 (12.7, 25.6) | <0.001 |
| **D-dimer (**μg/mL), median (IQR) | 7.8 (3.9, 17.2) | 38.8 (28.9, 65.5) | 12.7 (8.9, 19.0) | 3.1 (2.1, 4.1) | 7.9 (5.4, 11.8) | <0.001 |
| **Antithrombin (**%), median (IQR) | 60.0 (50.8, 69.0) | 58.2 (49.0, 67.5) | 51.0 (42.0, 59.1) | 60.9 (51.8, 71.0) | 63.2 (56.0, 70.9) | <0.001 |
| Lactate (mmol/L), median (IQR) | 2.9 (1.7, 5.7) | 4.0 (2.0, 7.6) | 3.8 (2.0, 7.9) | 2.6 (1.6, 4.8) | 2.5 (1.5, 4.8) | <0.001 |
| ISTH DIC score |  |  |  |  |  | <0.001 |
| 0 | 685 (18.7%) | 0 (0.0%) | 1 (2.0%) | 72 (53.7%) | 50 (23.1%) |  |
| 1 | 239 (6.5%) | 2 (3.3%) | 4 (7.8%) | 14 (10.4%) | 14 (6.5%) |  |
| 2 | 701 (19.2%) | 1 (1.6%) | 2 (3.9%) | 24 (17.9%) | 64 (29.6%) |  |
| 3 | 592 (16.2%) | 20 (32.8%) | 8 (15.7%) | 14 (10.4%) | 60 (27.8%) |  |
| 4 | 530 (14.5%) | 12 (19.7%) | 15 (29.4%) | 5 (3.7%) | 20 (9.3%) |  |
| 5 | 441 (12.1%) | 16 (26.2%) | 7 (13.7%) | 4 (3.0%) | 8 (3.7%) |  |
| 6 | 250 (6.8%) | 4 (6.6%) | 9 (17.6%) | 1 (0.7%) | 0 (0.0%) |  |
| 7 | 169 (4.6%) | 5 (8.2%) | 3 (5.9%) | 0 (0.0%) | 0 (0.0%) |  |
| 8 | 40 (1.1%) | 1 (1.6%) | 2 (3.9%) | 0 (0.0%) | 0 (0.0%) |  |
| ISTH DIC score ≥ 5 | 1430 (39.2%) | 471 (86.4%) | 436 (79.4%) | 611 (64.0%) | 820 (71.5%) | <0.001 |
| Management |  |  |  |  |  |  |
| rhTM | 969 (29.3%) | 251 (43.7%) | 225 (39.5%) | 205 (21.0%) | 288 (24.2%) | <0.001 |
| Vasopressor | 2789 (75.5%) | 497 (81.6%) | 524 (87.0%) | 765 (69.9%) | 1003 (72.2%) | <0.001 |
| Renal replacement therapy | 971 (26.3%) | 218 (35.8%) | 214 (35.5%) | 198 (18.1%) | 341 (24.6%) | <0.001 |
| Steroids | 894 (24.2%) | 173 (28.4%) | 170 (28.2%) | 237 (21.7%) | 314 (22.6%) | <0.001 |
| Intravenous immunoglobulin | 1088 (29.5%) | 204 (33.5%) | 217 (36.0%) | 279 (25.5%) | 388 (27.9%) | <0.001 |
| Antithrombin | 1092 (29.6%) | 249 (40.9%) | 259 (43.0%) | 248 (22.7%) | 336 (24.2%) | <0.001 |
| Outcomes |  |  |  |  |  |  |
| 28-day death | 753 (20.4%) | 153 (25.1%) | 178 (29.6%) | 180 (16.5%) | 242 (17.4%) | <0.001 |
| In-hospital death | 1186 (32.1%) | 213 (35.0%) | 287 (47.7%) | 286 (26.1%) | 400 (28.8%) | <0.001 |

Six coagulation markers (bold font) were used for clustering. Variables (red font) were potential confounders that were adjusted in a generalized estimating equation. **P* between clusters. Abbreviations: APACHE, Acute Physiology and Chronic Health Evaluation; DIC, disseminated intravascular coagulation; FDP, fibrinogen/fibrin degradation product; IQR, interquartile range; ISTH, International Society on Thrombosis and Haemostasis; PT-INR, prothrombin time-international normalized ratio; SIRS, Systemic Inflammatory Response Syndrome; rhTM: recombinant human thrombomodulin; SOFA, Sequential Organ Failure Assessment; WBC, white blood cells.

**Table S2. Unadjusted and adjusted risk difference between recombinant thrombomodulin and outcomes using divisive hierarchical clustering in derivation cohort.**

|  | Cluster dA-hc | Cluster d2-hc | Cluster d3-hc | Cluster d4-hc |
| --- | --- | --- | --- | --- |
| Unadjusted association (vs. non rhTM), risk difference (95%CI) | | | | |
| 28-Day death | -6.14 (-12.85–0.58) | -3.82 (-11.61–3.97) | 7.25 (1.42–13.08) | 1.01 (-4.15–6.17) |
| In-hospital death | -9.98 (-17.40– -2.56) | -6.59 (-15.21–2.02) | 6.84 (-0.13–13.81) | -1.73 (-7.90–4.44) |
| Adjusted association (vs. non rhTM), risk difference (95%CI) | | | | |
| 28-Day death | -5.16 (-11.85–1.53) | -3.73 (-10.76–3.30) | 3.68 (-1.78–9.15) | 0.47 (-4.33–5.27) |
| In-hospital death | -9.01 (-16.22–-1.80) | -6.52 (-14.08–1.04) | 1.06 (-5.29–7.40) | -2.91 (-8.64–2.83) |

In cluster dA-hc, rhTM was associated with better clinical outcomes (adjusted risk difference for 28-day mortality: -5.2% [95%CI, -11.9%–1.5%]; adjusted risk difference for in-hospital mortality -9.0% [95%CI, -16.2%–-1.8%]). Abbreviations: rhTM, recombinant human thrombomodulin.

**Table S3. Adjusted odds ratios between recombinant thrombomodulin and outcomes in derivation and validation cohorts using Bayesian regression.**

|  | Cluster dA | Cluster dB | Cluster dC | Cluster dD | |
| --- | --- | --- | --- | --- | --- |
| Adjusted association (vs. non rhTM use), odds ratio (95%CI) | | | | | |
| 28-Day death | -17.8 (-28.7–-6.9) | 0.7 (-7.1–8.6) | -3.1 (-8.3–2.1) | -0.7 (-4.5–6.0) | |
| In-hospital death | -17.7 (-27.6–-7.8) | 0.2 (-7.9–8.3) | -10.2 (-15.9–-4.6) | -1.3 (-7.6–4.9) | |
|  | Cluster vA | Cluster vB | Cluster vC | Cluster vD | |
| Adjusted association (vs. non rhTM use), odds ratio (95%CI) | | | | | |
| 28-Day death | 0.08 (0.01-0.43) | 0.66 (0.22-2.03) | 0.89 (0.37-1.95) | 0.72 (0.18-2.48) | |
| In-hospital death | 0.08 (0.01-0.36) | 0.65 (0.21-1.80) | 0.80 (0.38-1.67) | 1.00 (0.29-2.92) | |
| Results are shown as beta coefficients with intervals that have 95% chance of containing the value if all assumptions made for the model hold: 95%CI). Odds ratios with 95% CI are displayed for simplicity. Abbreviations: rhTM, recombinant human thrombomodulin. | | | | |  |

Results are shown as beta coefficients with 95% credible intervals. For simplicity, we displayed the odds ratio with a 95% credible interval (i.e., the interval that has a 95% chance of containing the value if all assumptions made for the model hold).

**Table S4. Characteristics of patients in the validation cohort based on clusters determined using k-means clustering.**

|  | Overall | Cluster vA | Cluster vB | Cluster vC | Cluster vD | *p* |
| --- | --- | --- | --- | --- | --- | --- |
| Variables | n = 1184 | n = 108 | n = 185 | n = 384 | n = 507 |  |
| Age, median (IQR) | 73.0 (64.0, 81.0) | 73.0 (64.0, 82.0) | 71.0 (64.0, 79.0) | 75.0 (66.0, 83.0) | 72.0 (62.0, 81.0) | 0.003 |
| Sex, female | 465 (39.3%) | 45 (41.7%) | 80 (43.2%) | 157 (40.9%) | 183 (36.1%) | 0.26 |
| Body weight kg, median (IQR) | 55.0 (47.0, 65.0) | 53.0 (46.5, 60.5) | 55.0 (47.3, 63.0) | 54.0 (46.0, 64.8) | 56.0 (48.2, 66.0) | 0.12 |
| CCI, median (IQR) | 1.0 (0.0, 2.0) | 2.0 (0.0, 3.0) | 1.0 (0.0, 3.0) | 1.0 (0.0, 2.0) | 1.0 (0.0, 2.0) | 0.009 |
| Infection site |  |  |  |  |  | <0.001 |
| Catheter-related | 22 (1.9%) | 6 (5.6%) | 0 (0.0%) | 11 (2.9%) | 5 (1.0%) |  |
| Bone/soft tissue | 138 (11.7%) | 7 (6.5%) | 26 (14.1%) | 46 (11.9%) | 59 (11.7%) |  |
| Cardiovascular | 16 (1.4%) | 5 (4.6%) | 0 (0.0%) | 7 (1.8%) | 4 (0.8%) |  |
| Central nervous system | 23 (1.9%) | 2 (1.9%) | 2 (1.1%) | 8 (2.1%) | 11 (2.2%) |  |
| Urinary tract | 218 (18.4%) | 31 (28.7%) | 25 (13.5%) | 82 (21.4%) | 80 (15.8%) |  |
| Lung/thoracic | 367 (31.0%) | 19 (17.6%) | 46 (24.9%) | 95 (24.7%) | 207 (40.8%) |  |
| Abdomen | 311 (26.3%) | 25 (23.1%) | 71 (38.4%) | 106 (27.6%) | 109 (21.5%) |  |
| Other/unknown | 89 (7.5%) | 13 (20.1%) | 15 (8.1%) | 29 (7.5%) | 32 (6.3%) |  |
| APACHE2, median (IQR) | 22.0 (16.5, 29.0) | 27.0 (22.0, 32.0) | 26.0 (20.0, 32.0) | 23.0 (17.0, 29.0) | 19.0 (14.0, 26.0) | <0.001 |
| SIRS score, median (IQR) | 3.0 (2.3, 4.0) | 3.0 (3.0, 4.0) | 3.0 (2.0, 3.0) | 3.0 (3.0, 4.0) | 3.0 (2.0, 4.0) | 0.11 |
| SOFA scores | 8.4 (5.6, 11.0) | 11.0 (9.0, 13.5) | 11.0 (8.0, 13.0) | 9.0 (7.0, 11.0) | 6.0 (4.0, 9.0) | <0.001 |
| Laboratory data |  |  |  |  |  |  |
| WBC (10^3^/μL), median (IQR) | 11.3 (58.8, 177.3) | 10.8 (6.3, 20.0) | 8.8 (3.7, 15.9) | 11.3 (5.1, 17.5) | 12.6 (6.7, 18.0) | 0.002 |
| **Platelets (**10^3^/μL), median (IQR) | 144.0 (90.0, 220.0) | 68.0 (40.5, 120.5) | 101.0 (59.0, 155.0) | 122.5 (82.0, 176.5) | 198.0 (137.0, 269.0) | <0.001 |
| **PT-INR**, median (IQR) | 1.2 (1.1, 1.4) | 1.5 (1.3, 1.7) | 1.6 (1.4, 2.2) | 1.2 (1.1, 1.4) | 1.2 (1.1, 1.3) | <0.001 |
| **Fibrinogen (**mg/mL), median (IQR) | 447.0 (327.5, 563.0) | 276.8 (154.0, 381.0) | 302.0 (222.0, 394.0) | 465.5 (379.8, 570.4) | 505.0 (416.6, 625.4) | <0.001 |
| **FDP (**μg/mL), median (IQR) | 21.8 (11.0, 46.7) | 120.7 (93.0, 245.4) | 19.8 (9.2, 37.6) | 41.8 (28.4, 62.4) | 11.7 (7.7, 17.1) | <0.001 |
| **D-dimer (**μg/mL), median (IQR) | 9.2 (4.3, 21.9) | 60.1 (39.5, 105.6) | 8.5 (3.7, 15.3) | 19.6 (13.2, 29.0) | 4.6 (2.7, 7.3) | <0.001 |
| **Antithrombin (**%), median (IQR) | 67.0 (55.0, 75.9) | 55.0 (49.0, 65.6) | 45.0 (35.0, 54.6) | 66.0 (58.0, 72.1) | 73.8 (67.0, 81.4) | <0.001 |
| Lactate (mmol/L), median (IQR) | 3.0 (1.8, 5.2) | 4.6 (2.9, 7.3) | 4.1 (2.3, 6.5) | 3.3 (2.0, 5.6) | 2.3 (1.5, 3.6) | <0.001 |
| ISTH DIC score |  |  |  |  |  | <0.001 |
| 0 | 122 (10.3%) | 0 (0.0%) | 2 (1.1%) | 0 (0.0%) | 120 (23.7%) |  |
| 1 | 108 (9.1%) | 0 (0.0%) | 6 (3.2%) | 2 (0.5%) | 100 (19.7%) |  |
| 2 | 240 (20.3%) | 0 (0.0%) | 34 (18.4%) | 26 (6.8%) | 180 (35.5%) |  |
| 3 | 260 (22.0%) | 7 (6.5%) | 42 (22.7%) | 134 (34.9%) | 77 (15.2%) |  |
| 4 | 232 (19.6%) | 27 (25.0%) | 43 (23.2%) | 136 (35.4%) | 26 (5.1%) |  |
| 5 | 147 (12.4%) | 37 (34.3%) | 37 (20.0%) | 69 (18.0%) | 4 (0.8%) |  |
| 6 | 53 (4.5%) | 22 (20.4%) | 17 (9.2%) | 14 (3.6%) | 0 (0.0%) |  |
| 7 | 19 (1.6%) | 14 (13.0%) | 2 (1.1%) | 3 (0.8%) | 0 (0.0%) |  |
| 8 | 3 (0.3%) | 1 (0.9%) | 2 (1.1%) | 0 (0.0%) | 0 (0.0%) |  |
| ISTH DIC score ≥ 5 | 454 (38.3%) | 101 (93.5%) | 101 (54.6%) | 222 (57.8%) | 30 (5.9%) |  |
| Management |  |  |  |  |  |  |
| rhTM | 242 (21.2%) | 44 (44.4%) | 54 (31.2%) | 98 (26.3%) | 46 (9.3%) | <0.001 |
| Vasopressor | 749 (63.3%) | 77 (71.3%) | 147 (79.5%) | 267 (69.5%) | 258 (50.9%) | <0.001 |
| Renal replacement therapy | 98 (8.3%) | 16 (14.8%) | 28 (15.1%) | 39 (10.2%) | 15 (3.0%) | <0.001 |
| Steroids | 345 (29.1%) | 48 (44.4%) | 81 (43.8%) | 112 (29.2%) | 104 (20.5%) | <0.001 |
| Intravenous immunoglobulin | 94 (7.9%) | 18 (16.7%) | 26 (14.1%) | 42 (10.9%) | 8 (1.6%) | <0.001 |
| Antithrombin | 131 (11.1%) | 22 (20.4%) | 35 (18.9%) | 52 (13.5%) | 22 (4.3%) | <0.001 |
| Outcomes |  |  |  |  |  |  |
| 28-day death | 269 (23.4%) | 24 (24.5%) | 67 (38.7%) | 70 (18.8%) | 55 (11.1%) | <0.001 |
| In-hospital death | 216 (19.0%) | 28 (28.0%) | 81 (46.3%) | 92 (24.6%) | 68 (13.6%) | <0.001 |

Six coagulation markers (bold font) were used for clustering. Variables (red font) were potential confounders that were adjusted in a generalized estimating equation. Abbreviations: APACHE, Acute Physiology and Chronic Health Evaluation; CCI, Charlson Comorbidity Index; DIC, disseminated intravascular coagulation; FDP, fibrinogen/fibrin degradation product; IQR, interquartile range; ISTH, International Society on Thrombosis and Haemostasis; PT-INR, prothrombin time-international normalized ratio; SIRS, Systemic Inflammatory Response Syndrome; rhTM: recombinant human thrombomodulin; SOFA, Sequential Organ Failure Assessment; WBC, white blood cells.

**Table S5. Unadjusted and adjusted risk difference between recombinant thrombomodulin use and secondary outcomes in the delivation cohort.**

| Outcomes | Cluster vA | *p*-value | Cluster vB | *p*-value | Cluster vC | *p*-value | Cluster vD | *p*-value |
| --- | --- | --- | --- | --- | --- | --- | --- | --- |
| Risk difference, % (95%CI) | | | | | | | | |
| Unadjusted association (vs. non rhTM use) | | | | | | | | |
| Ventilator-free days | 6.2 (1.6 to 10.7) | 0.008 | -2.7 (-6.5 to 1.1) | 0.16 | -4.4 (-7.1 to -1.7) | 0.002 | -4.2 (-7.5 to -0.90) | 0.013 |
| ICU-free days | 2.7 (-1.3 to 6.8) | 0.19 | -3.5 (-7.2 to 0.12) | 0.058 | -3.4 (-5.8 to -1.07) | 0.004 | -2.1 (-5.0 to 0.82) | 0.16 |
| Discharge to home, % | 0.02 (-0.19 to 0.22) | 0.88 | -0.08 (-0.27 to 0.12) | 0.45 | -0.06 (-0.19 to 0.08) | 0.40 | -0.13 (-0.31 to 0.03) | 0.12 |
| Adjusted association (vs. non rhTM use) | | | | | | | | |
| Ventilator-free days | 6.7 (0.76 to 12.7) | 0.027 | 1.5 (-4.3 to 7.3) | 0.62 | -4.8 (-8.6 to -0.93) | 0.015 | 0.27 (-4.1 to 4.6) | 0.91 |
| ICU-free days | 0.55 (-4.6 to 5.7) | 0.83 | -1.1 (-7.2 to 5.1) | 0.74 | -1.3 (-4.6 to 2.1) | 0.46 | -0.63 (-4.4 to 3.2) | 0.75 |
| Discharge to home, % | 0.01 (-0.25 to 0.27) | 0.93 | 0.11 (-0.21 to 0.44) | 0.51 | -0.002 (-0.19 to 0.19) | 0.99 | -0.01 (-0.23 to 0.21) | 0.92 |
| Abbreviations: rhTM, recombinant human thrombomodulin | | | | | | | | |

The adjusted variables were age, sex, comorbidities, SOFA scores, and in-hospital management, including renal replacement therapy, and treatment with steroids, intravenous immunoglobulin, antithrombin, and vasopressors.

**Table S6. Risk difference between recombinant thrombomodulin use and outcomes, adjusted variables including Acute Physiology and Chronic Health Evaluation scores and source of infection.**

| **Outcomes** | Cluster vA | *p*-value | Cluster vB | *p*-value | Cluster vC | *p*-value | Cluster vD | *p*-value |
| --- | --- | --- | --- | --- | --- | --- | --- | --- |
| Associations in the validation cohorts, risk difference, % (95%CI) | | | | | | | | |
| Adjusted association (vs. non rhTM use) | | | | | | | | |
| 28-day death | -0.24 (-0.45 to -0.02) | 0.029 | 0.02(-0.22 to 0.27) | 0.85 | 0.01 (-0.12 to 0.15) | 0.84 | -0.06 (-0.19 to 0.06) | 0.33 |
| In-hospital death | -0.26 (-0.47 to -0.04) | 0.02 | 0.03 (-0.22 to 0.28) | 0.81 | -0.01 (-0.16 to 0.14) | 092 | 0.01 (-0.13 to 0.15) | 0.89 |
| Abbreviations: rhTM, recombinant human thrombomodulin | | | | | | | | |

The adjusted variables were age, sex, Acute Physiology and Chronic Health Evaluation (APACHE II) scores, source of infection, and in-hospital management, including renal replacement therapy, and treatment with steroids, intravenous immunoglobulin, antithrombin, and vasopressors.
