# Supplementary material for: Coagulation phenotypes in sepsis and effects of recombinant human thrombomodulin: an analysis of three multicentre observational studies
Source: Crit Care. 2021 Mar 19;25:114. doi: 10.1186/s13054-021-03541-5 (PMC7978458; doi:10.1186/s13054-021-03541-5)
Supplement: Supplementary file 3 — Additional file 3. Supplemental Figures. [file 13054_2021_3541_MOESM3_ESM.docx]

**Figure S1. The distribution of the proportion of patients treated with recombinant thrombomodulin at the various institutes**

Each bar indicates the number of the institutes at which the indicated percentage of patients were treated using rhTM i.e., the bar between 10 and 20 represents the number of institutes, in which the proportion of patients treated with rhTM was between 10% and 20%. a, in the derivation cohort; b, in the validation cohort

rhTM, recombinant human thrombomodulin

**Figure S2. Consensus matrix heatmaps based on number of clusters**


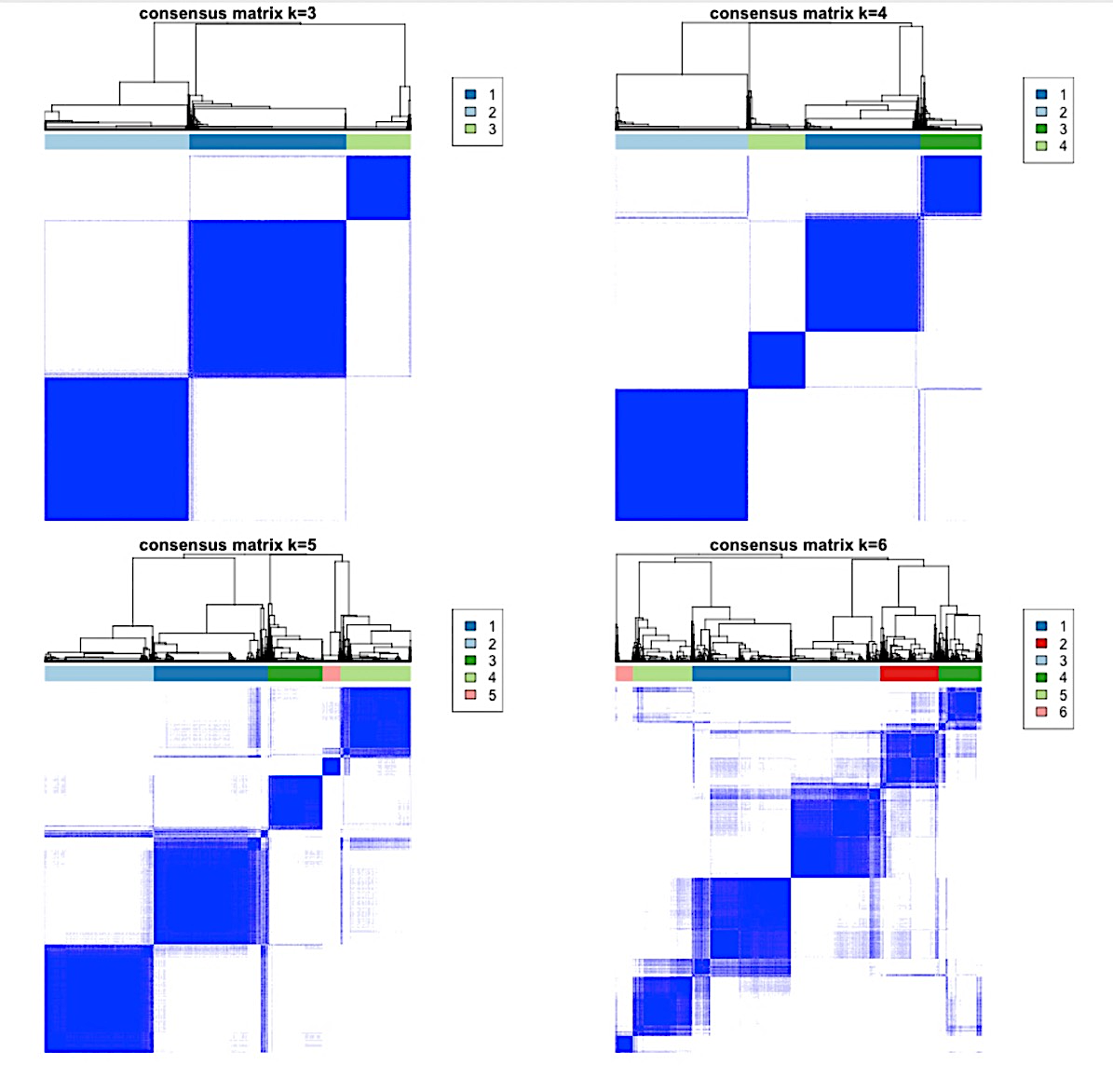


Heatmaps indicate that patients with sepsis comprise four clusters with relatively clear boundaries.

**Figure S3. Elbow method**


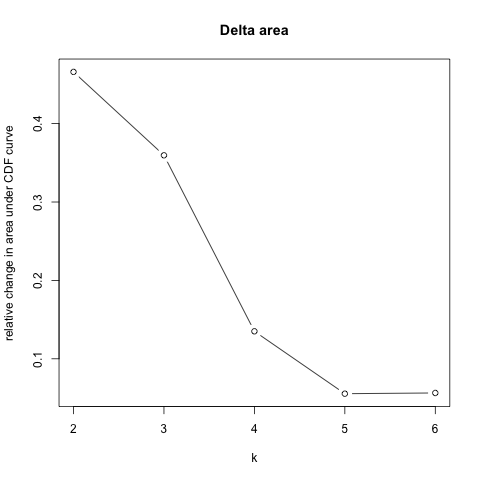


**Relative change in area under CDF curve**

**Delta area**

**k**

Elbow method shows ratios (%) of variance as functions of the number of clusters. Several clusters are selected such that adding another cluster will not greatly improve the modeling data. Relative change in area under cumulative density function (CDF) was highest for four clusters.

**Figure S4. Consensus cumulative distribution function plot**


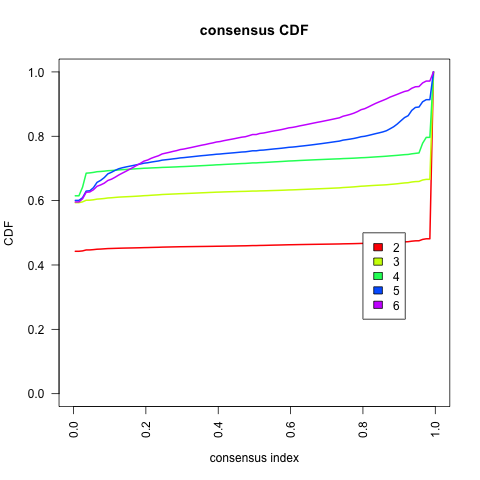


**Cumulative distribution function**

**Consensus CDF**

**Consensus index**

This plot represents the cumulative density function of consensus values for given number of clusters. Optimal number of clusters is represented by CDF plot with first step close to zero followed by plateau until reaching second step close to one. This consensus CDF plot implies that four or five clusters are optimal.

**Figure S5. Cluster consensus plot**

This plots represents consensus values of all pairs within each cluster. Clusters with higher cluster consensus (> 0.8) for all clusters are preferred. These results imply the absence of clearly separated clusters.

**Figure S6. Cluster dendrogram using divisive hierarchical clustering**


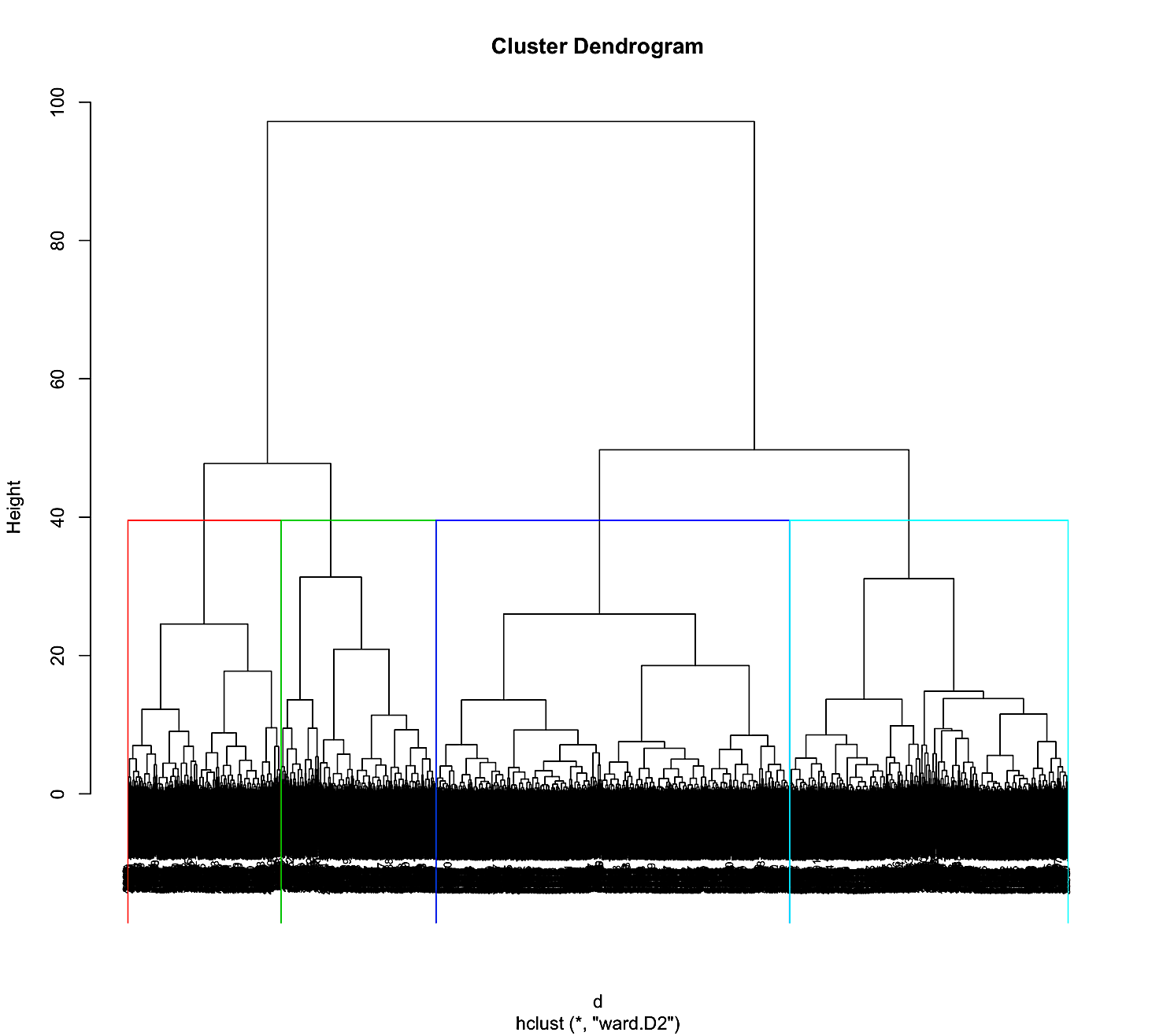


Hierarchical relationships between patients in terms of coagulation. Patients were divided into two large clusters that were further subdivided into two.

**Figure S7. Elbow method for identifying the optimal number of clusters through divisive hierarchical clustering**


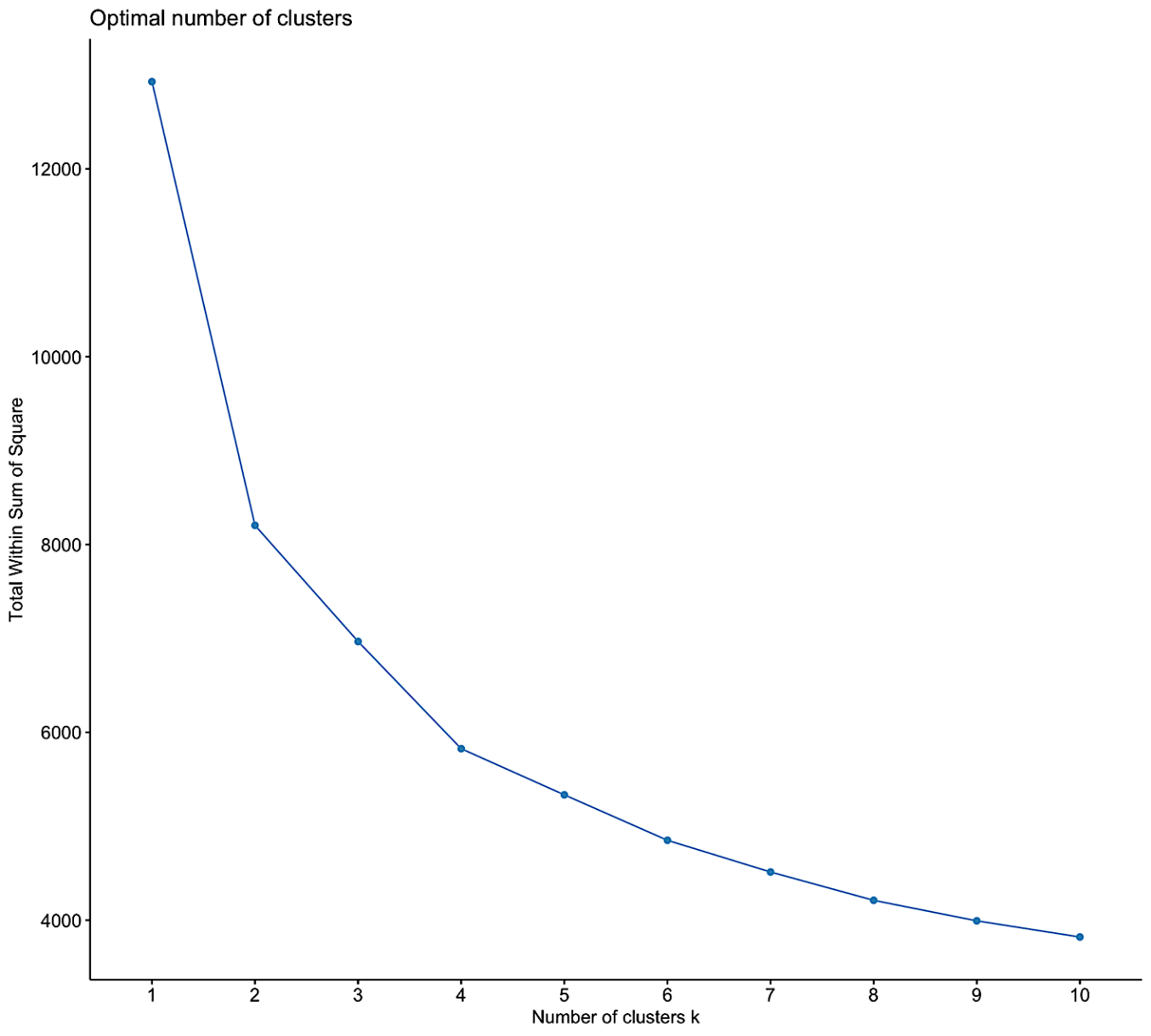


**Optimal number of clusters**

**Total within sum of square**

**Number of clusters k**

Optimal number of clusters is determined using divisive hierarchical clustering. Several clusters are selected such that adding another cluster will not greatly improve modeling data. Relative change in area under cumulative density function (CDF) is highest for two or four clusters.

**Figure S8. Gap statistic method to identify optimal number of clusters using divisive hierarchical clustering**


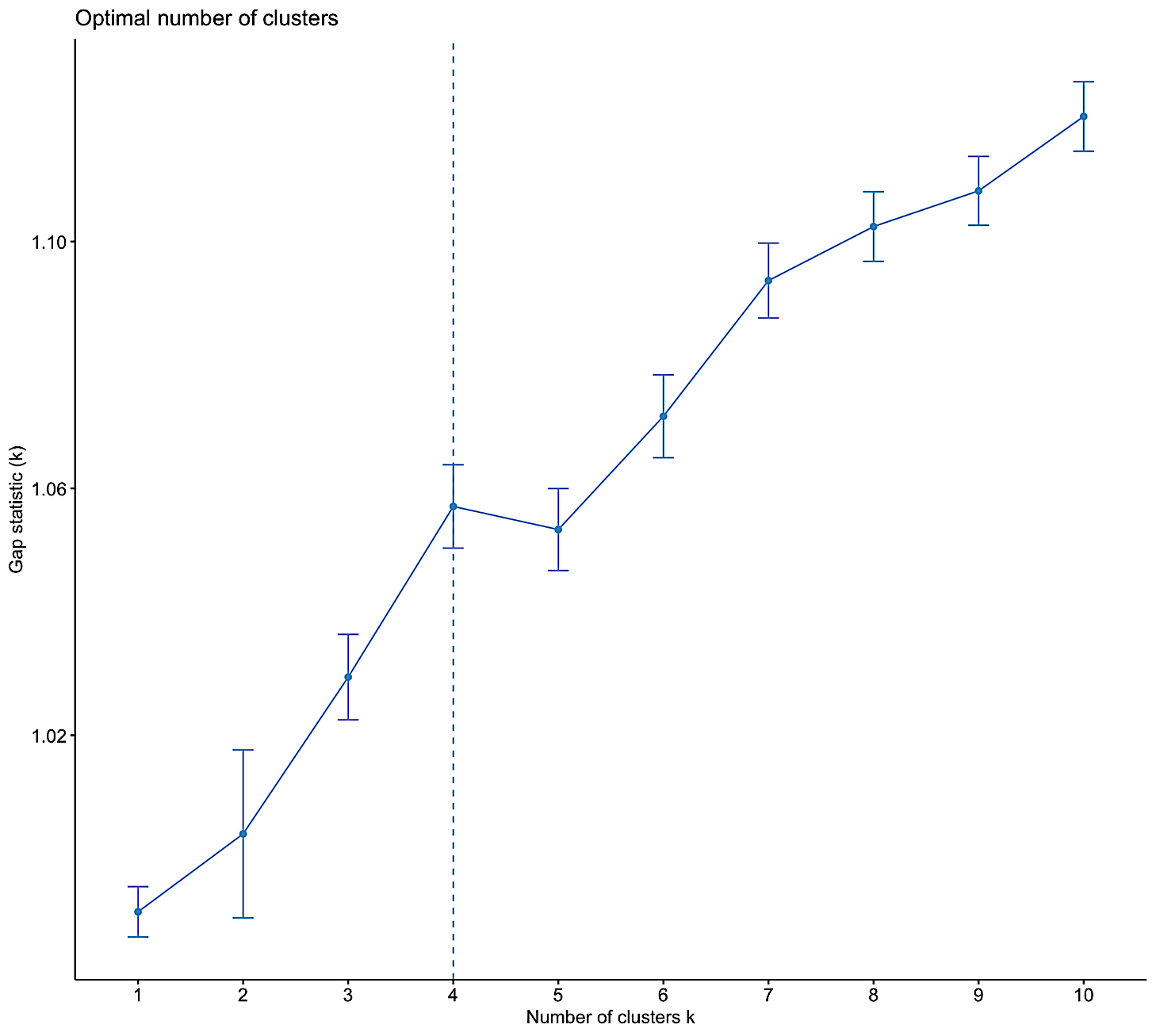


Total intra-cluster variation for different k values with their predicted values under null reference distribution of the data. Estimated optimal clusters will be the ones with values that yield the largest gap statistic. The optimal number of clusters is four.

**Figure S9. Bayesian heterogeneity of the rhTM effects in the validation cohort**

**
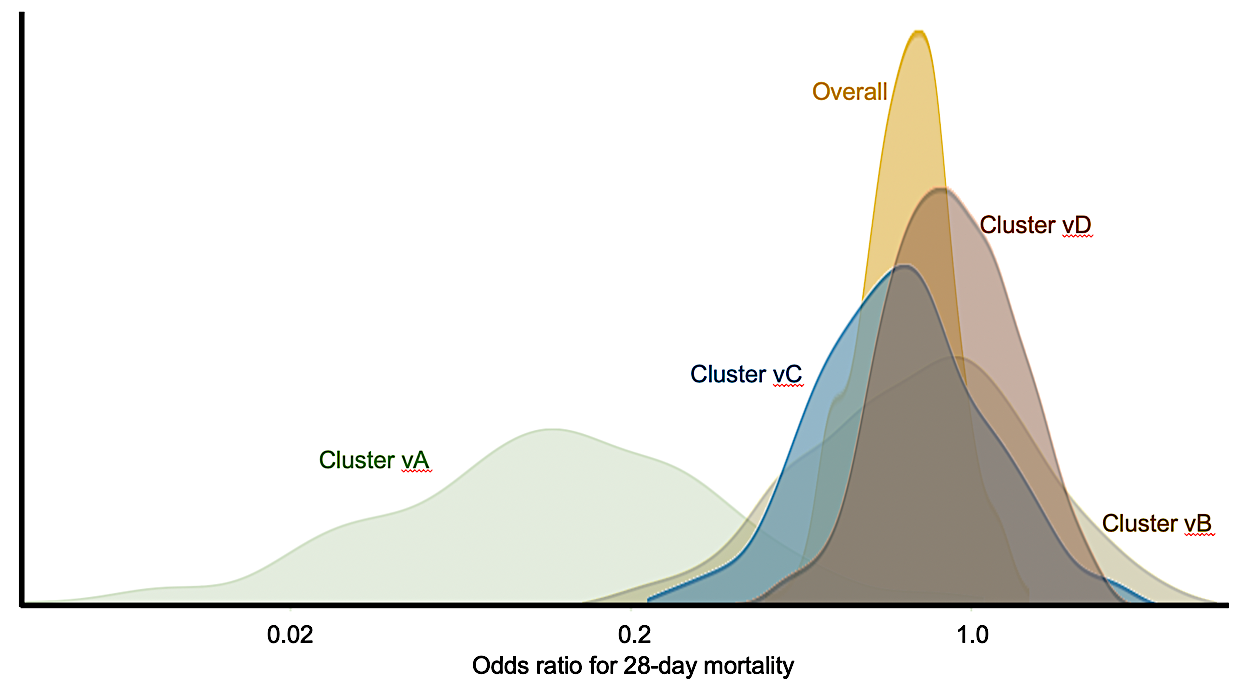
**

X-axis, odds ratio; Y-axis, density. Results indicate significant association between rhTM and 28-day mortality in the validation cohort, but not when considering the overall data.
